# Patient-Derived Organoid Modeling of Glypican-3 CAR-T Responses in Hepatocellular Carcinoma

**DOI:** 10.3390/cells15090799

**Published:** 2026-04-28

**Authors:** Bohan Zhang, Yun Deng, Mingshan Zhou, Junfei Chen, Jiawen Wu, Xiaofeng Lian, Miaoxin Zhu, Min Zhou, Jie Cao

**Affiliations:** 1 State Key Laboratory of Systems Medicine for Cancer, Shanghai Cancer Institute, Renji Hospital, Shanghai Jiao Tong University School of Medicine, Shanghai 200032, China; bohan6309@sjtu.edu.cn (B.Z.); 18111520013@fudan.edu.cn (M.Z.); jf_chen@zju.edu.cn (J.C.); wjw-2001@sjtu.edu.cn (J.W.); lianxiaofeng@sjtu.edu.cn (X.L.); zhumiaoxin@renji.com (M.Z.); 2 Department of Radiation Oncology, Fudan University Shanghai Cancer Center, Fudan University, Shanghai 200032, China; 15111230011@fudan.edu.cn; 3 Department of Thoracic Surgery, Shanghai Chest Hospital, Shanghai Jiao Tong University School of Medicine, Shanghai 200030, China; 4 Department of Liver Surgery, Renji Hospital, Shanghai Jiao Tong University School of Medicine, 160 Pujian Road, Shanghai 200127, China

**Keywords:** hepatocellular carcinoma, patient-derived organoids, chimeric antigen receptor T cell, glypican-3

## Abstract

Glypican-3 (GPC3)-targeted chimeric antigen receptor T (CAR-T) cell therapy is a promising approach for hepatocellular carcinoma (HCC), but marked interpatient variability and antigen heterogeneity limit its broader application. Here, we established a patient-derived organoid (PDO)-based platform to functionally evaluate autologous GPC3-targeted CAR-T cell activity in HCC. HCC PDOs preserved key histologic features and heterogeneous GPC3 expression patterns of the original tumors. In co-culture assays, CAR-T cell cytotoxicity was associated with GPC3 expression levels and was accompanied by IFN-γ and IL-2 release, supporting the feasibility of using PDOs for functional assessment of CAR-T cell sensitivity. We further found that matrix conditions strongly influenced organoid architecture, viral transduction, CAR-T cell infiltration, and killing efficiency, with lower Matrigel concentrations providing a more permissive setting for functional assessment. Importantly, in GPC3-low PDOs, pretreatment with the DNA methyltransferase inhibitor 5-azacytidine (5-AZA) reduced DNA methyltransferase 3 alpha (DNMT3A) expression, increased surface GPC3 expression, and significantly enhanced CAR-T-mediated cytotoxicity. Together, these findings provide proof-of-concept evidence supporting the use of HCC PDOs as a patient-derived platform for modeling selected determinants of GPC3-targeted CAR-T cell activity and for exploring combination strategies to improve therapeutic efficacy.

## 1. Introduction

Hepatocellular carcinoma (HCC) remains a leading cause of cancer-related mortality worldwide, with limited therapeutic options for patients with advanced disease [1,2]. Although recent advances in systemic therapies, including immune checkpoint inhibitors, have improved outcomes in a subset of patients, durable responses remain uncommon. Adoptive cell therapies, particularly chimeric antigen receptor T cell (CAR-T) therapy, have demonstrated remarkable success in hematologic malignancies and are being actively explored for solid tumors such as HCC [3,4,5]. Among candidate targets, glypican-3 (GPC3), a membrane-bound oncofetal proteoglycan highly expressed in a subset of HCCs, has emerged as a promising antigen for CAR-T-based strategies. Early-phase clinical studies have demonstrated the feasibility of GPC3-targeted CAR-T therapy; however, substantial interpatient variability in efficacy highlights the need for improved strategies to identify responsive patients, guide personalized treatment selection, and overcome resistance mechanisms [4,6,7].

A major barrier to effective CAR-T therapy in solid tumors is antigen heterogeneity. In HCC, GPC3 expression varies widely both between patients and within individual tumors, leading to incomplete tumor targeting and potential immune escape. In addition, the complex tumor microenvironment (TME), including physical barriers imposed by extracellular matrix components and the presence of immunosuppressive cells, further limits CAR-T cell infiltration and effector function. Together, these factors contribute to the limited and inconsistent clinical efficacy observed in current trials. Therefore, there is a critical need for physiologically relevant preclinical models that can capture patient-specific tumor characteristics, including antigen heterogeneity, and enable functional evaluation of CAR-T cell activity.

Patient-derived organoids (PDOs) have emerged as a powerful platform for modeling human cancers in vitro. These three-dimensional cultures preserve key tumor-intrinsic features, including genomic alterations, histologic architecture, and cellular heterogeneity, and have been applied to predict responses to chemotherapy and targeted therapies across multiple tumor types [8,9,10,11]. In HCC, PDOs can be established from resected tumor tissues and have been shown to retain important molecular and phenotypic characteristics of the original tumors [12,13]. However, the application of PDOs to evaluate adoptive cell therapies remains relatively underexplored. In particular, it is unclear whether PDOs can preserve therapeutically relevant antigen heterogeneity, support functional interactions with autologous immune cells, and serve as a platform for exploring strategies to overcome antigen-dependent resistance.

In addition to tumor-intrinsic factors, experimental conditions within organoid systems may substantially influence the apparent efficacy of immune effector cells. The extracellular matrix, commonly modeled using Matrigel, not only supports three-dimensional organoid growth but also affects cell–cell interactions, immune-cell infiltration, and the diffusion of nutrients and signaling molecules. Variations in matrix composition and stiffness may, therefore, alter CAR-T cell access to tumor cells and confound functional readouts [14,15,16]. Systematic evaluation of these parameters is essential to establish a robust and reproducible platform for assessing CAR-T cell activity in PDO models.

In antigen-low tumors, pharmacologic modulation of target antigen expression may provide a potential strategy to enhance CAR-T cell recognition and cytotoxicity [17]. DNA methylation is an important epigenetic mechanism regulating gene expression, and DNA methyltransferase inhibitors may restore or increase the expression of selected tumor-associated antigens [18,19]. This rationale is particularly relevant to HCC, as recent multi-omic evidence supports an association between DNA methylation and GPC3 expression regulation [19].

Here, we established a biobank of patient-derived HCC organoids and investigated whether these models preserve the heterogeneous expression of GPC3 observed in primary tumors. We then developed and optimized an autologous co-culture system to functionally evaluate GPC3-targeted CAR-T cell activity, focusing on key parameters such as matrix conditions and effector-to-target (E:T) ratios. Using this platform, we quantified CAR-T cell infiltration, cytotoxicity, and cytokine production, and examined the relationship between antigen abundance and therapeutic response. Finally, we explored a rational sensitization strategy for antigen-low tumors by pharmacologically modulating GPC3 expression using a DNA methyltransferase inhibitor. Together, our study provides a proof-of-concept patient-derived platform for functionally evaluating GPC3-targeted CAR-T cell activity and exploring combination strategies to address antigen heterogeneity in HCC.

## 2. Materials and Methods

### 2.1. Patient Samples and Cohort Overview

Resected HCC specimens and matched peripheral blood samples were collected from patients undergoing surgery. A total of 19 tumor samples were processed for PDO establishment, of which 16 successfully generated organoid cultures. Among these, 3 PDO lines were used for histologic and molecular characterization, 3 matched PDO lines were used for CAR-T co-culture assays, and 4 low-GPC3 PDO lines were used for 5-azacytidine (5-AZA, Selleck, Shanghai, China) sensitization experiments. Tumor tissues from all 19 cases were included in the immunohistochemistry (IHC) analysis. Additional public datasets were used for correlative analyses as described below.

### 2.2. Isolation and Primary Culture of HCC Organoids

HCC PDOs culture media (Table S1) were prepared according to previously published protocols with modifications [12,13,20,21]. Resected HCC tumor tissues were collected and stored in pre-cooled tissue preservation solution at 4 °C. After washing with PBS, the tumor tissues were cut into approximately 1 mm^3^ pieces and digested with a tissue dissociation enzyme mixture containing collagenase I (0.25 mg/mL; Sigma, St. Louis, MO, USA) and collagenase IV (1.5 mg/mL; Sigma, St. Louis, MO, USA) in a 37 °C water bath for 20–40 min. Digestion was stopped once tumor cell clusters were observed to dissociate under the microscope. The suspension was then mixed with 3 volumes of PBS and centrifuged at 400× *g* for 7 min. The collected cells were suspended in HCC PDOs culture media mixed with Matrigel (Huayi Regeneration, Guangzhou, China) at different concentrations, and 10 µL droplets containing 1 × 10^4^ cells were seeded into 96-well round-bottom plates. After gelation at 37 °C for 20 min, PDO culture media were added, and the cultures were maintained until further analysis.

### 2.3. Immunofluorescence Staining of HCC Organoids

HCC organoids were retrieved from the Matrigel and gently washed two to three times with PBS. The organoids were then fixed in 4% paraformaldehyde, embedded in paraffin, sectioned, and mounted onto glass slides. After deparaffinization in xylene, antigen retrieval was performed, followed by blocking with 10% goat serum for 1 h at room temperature. Primary antibodies against GPC3, Hep Par-1, and Ki-67 (Santa Cruz Biotechnology, Dallas, TX, USA) were diluted at 1:200–1:500 and incubated overnight at 4 °C. On the following day, organoids were washed three times with PBS and incubated with appropriate fluorophore-conjugated secondary antibodies (1:500) for 1 h at room temperature in the dark. Nuclei were stained with DAPI (Roche Diagnostics GmbH, Mannheim, Germany) for 10 min, followed by additional PBS washes. Images were acquired using a confocal microscope (Mica Microhub, Leica microsystems, Wetzlar, Germany).

### 2.4. Immunohistochemistry of HCC Tissues

HCC tissues were fixed in 4% paraformaldehyde at 4 °C overnight, dehydrated through graded ethanol, cleared in xylene, and embedded in paraffin. Sections of 3–4 µm thickness were cut and baked at 65 °C for 1 h. After deparaffinization in xylene and rehydration through graded ethanol, endogenous peroxidase activity was blocked with 3% H_2_O_2_ for 10–15 min at room temperature. Heat-induced antigen retrieval was performed in EDTA buffer (pH 9.0), followed by blocking with 5% normal goat serum for 20–30 min. Primary anti-GPC3 antibody was added and incubated overnight at 4 °C. After three washes with PBS the next day, HRP-conjugated secondary antibodies were applied and incubated for 60 min at room temperature. Signals were developed with DAB, and nuclei were counterstained with hematoxylin. Sections were dehydrated, cleared, and mounted with neutral resin. Images were acquired using a NanoZoomer S360MD slide scanner system (Hamamatsu, Shizuoka, Japan). GPC3 expression was scored semi-quantitatively based on the percentage of positive tumor cells per field (average of multiple fields): − (0%), + (1–19%), ++ (20–50%), and +++ (>50%).

### 2.5. Flow-Cytometric Analysis of GPC3 Expression in HCC Organoids

HCC organoids were collected and digested with 0.25% trypsin in a 37 °C water bath for 5–10 min to obtain a single-cell suspension. After neutralization with Dulbecco’s Modified Eagle Medium (DMEM) containing 10% FBS, cells were centrifuged at 400× *g* for 7 min and washed once with PBS. Cells were then stained with an anti-GPC3 antibody for 1 h at room temperature. After washing with PBS, cells were incubated with a PE-conjugated goat anti-mouse IgG (BD Biosciences, San Jose, CA, USA) for 30 min at room temperature and then analyzed by flow cytometry. Data were processed using FlowJo version 10.10.0.

### 2.6. Viral Transduction of HCC Organoids and Measurement of Luciferase Activity

HCC organoids were collected and dissociated into single cells, then resuspended in Matrigel at final concentrations of 2 mg/mL, 3.3 mg/mL, 5 mg/mL, or 6.7 mg/mL. A 10 µL droplet containing 1 × 10^4^ cells was seeded into each well of a 96-well round-bottom plate, followed by addition of 200 µL PDO media containing lentivirus at multiplicities of infection (MOIs) 30, 15, 5, or 1. After 72 h, fluorescence images were acquired using a fluorescence microscope. The proportion of GFP-positive cells, the integrated fluorescence intensity (ImageJ software V1.8.0.112; National Institutes of Health, Bethesda, MD, USA), and luciferase activity were quantified to assess transduction efficiency.

### 2.7. CAR-T Cell Generation and the Assessment of CAR-T-Cell Cytotoxicity

The GPC3-targeted CAR consists of a humanized anti-GPC3 single-chain variable fragment, CD8α hinge and transmembrane domains, a CD28 intracellular domain, and a CD3ζ intracellular signaling domain, as previously described [22]. PBMCs were isolated from patient blood by density-gradient centrifugation using Ficoll-Paque (GE Healthcare, Chicago, IL, USA). T cells were stimulated with CD3/CD28 beads (Invitrogen, Carlsbad, CA, USA) at a bead-to-T cell ratio of 1:1 or with 2.5 μg/mL concanavalin A (ConA) for 48 h. After activation, cells were transduced with lentiviral vectors at an MOI of 5 by spinoculation (1200× *g*, 40 min) in 24-well plates pre-coated with RetroNectin (Takara Bio, Kyoto, Japan). Seven days after initial stimulation, Dynabeads were removed with a DynaMag-Spin magnet (Invitrogen, Carlsbad, CA, USA). The resulting CAR-T cells were cultured in AIM-V medium (Gibco, Waltham, MA, USA) supplemented with 5% AB-type human serum and 500 U/mL recombinant human IL-2 (Huaxin Biotech, Shanghai, China) at 37 °C in a humidified incubator with 5% CO_2_.

CAR-T cell cytotoxicity was assessed using PDO numbers, GFP fluorescence intensity, and luciferase-based reporter assays. Briefly, luciferase-GFP-expressing PDOs were seeded in 96-well round-bottom plates at a density of 1 × 10^4^ cells/well. Untransduced T (UT) cells or CAR-T cells were then added at different E:T ratios, including 1:3, 1:1, and 3:1. After 7 days of co-culture, residual PDOs were evaluated by measuring PDO numbers, GFP fluorescence intensity, or bioluminescence after the addition of D-luciferin potassium salt.

### 2.8. Cytokine Analysis

Supernatants were collected after 7 days of co-culture of CAR-T cells with HCC organoids. A 50 µL aliquot of supernatant was used for cytokine measurement. According to the manufacturer’s instructions, a cytometric bead array (CBA; BD Biosciences Pharmingen, San Diego, CA, USA) was used to quantify IFN-γ, TNF, IL-2, IL-4, IL-6, and IL-10 in the co-culture supernatants.

### 2.9. Correlation Analysis Between GPC3 Expression and Promoter DNA Methylation

GPC3 expression (RNA-seq) and promoter DNA methylation (HumanMethylation450 array) data for 371 HCC tumor samples were obtained from The Cancer Genome Atlas (TCGA) via the Genomic Data Commons (GDC) portal. Expression values were transformed to log2 (transcripts per million [TPM] + 1). Methylation levels were quantified by averaging the β values of all associated HM450 probes. Pearson correlation analysis was then performed to evaluate the relationship between GPC3 expression and promoter DNA methylation.

### 2.10. GPC3 Expression in HCC Tumors and Adjacent Normal Tissues

Proteomics data [23] were obtained from the International Cancer Proteogenome Consortium. GPC3 protein expression levels were quantified using Unshared Log Ratio values, calculated exclusively from unique peptides to ensure protein-specific quantification. Samples were annotated as tumor or adjacent normal tissues based on the corresponding clinical metadata.

### 2.11. Analysis of DNA Methyltransferase 3 Alpha (DNMT3A) Expression in HCC Organoids by Western Blotting

Three independent HCC PDOs were treated with 5 μmol/L 5-AZA or vehicle for 72 h. Total protein was extracted from PDOs using 2% SDS lysis buffer. Protein concentrations were determined using a BCA protein assay kit according to the manufacturer’s instructions. Equal amounts of protein were separated by SDS-PAGE and transferred onto membranes. After blocking, the membranes were incubated with the primary antibody (anti-DNMT3A, sc-373905, Santa Cruz Biotechnology, Santa Cruz, CA, USA; anti-β-actin, AF5003, Beyotime, Shanghai, China) overnight at 4 °C, followed by incubation with the corresponding secondary antibody for 1 h at room temperature. Protein bands were visualized using an enhanced chemiluminescence (ECL) detection system.

### 2.12. Statistical Analysis

Unless otherwise indicated, quantitative data are presented as mean ± SD from three technical replicate wells per condition. The number of independent PDO lines or independent experiments used for each analysis is specified in the corresponding figure legend or text. For experiments using multiple technical wells from the same PDO line, technical replicates were averaged before statistical comparison, where appropriate. One-way analysis of variance (ANOVA) was used for comparisons among multiple groups, and two-way ANOVA was applied to analyze CAR-T cell cytotoxicity, cytokine measurements, and CD4^+^ and CD8^+^ T cell infiltration. An unpaired two-tailed Student’s *t* test was used for comparisons between two groups. All statistical analyses were performed using GraphPad Prism version 10.1.2. A value of *p* < 0.05 was considered statistically significant.

## 3. Results

### 3.1. Establishment and Characterization of an HCC PDO-Based Platform for CAR-T Cell Efficacy Evaluation

**Figure 1:**
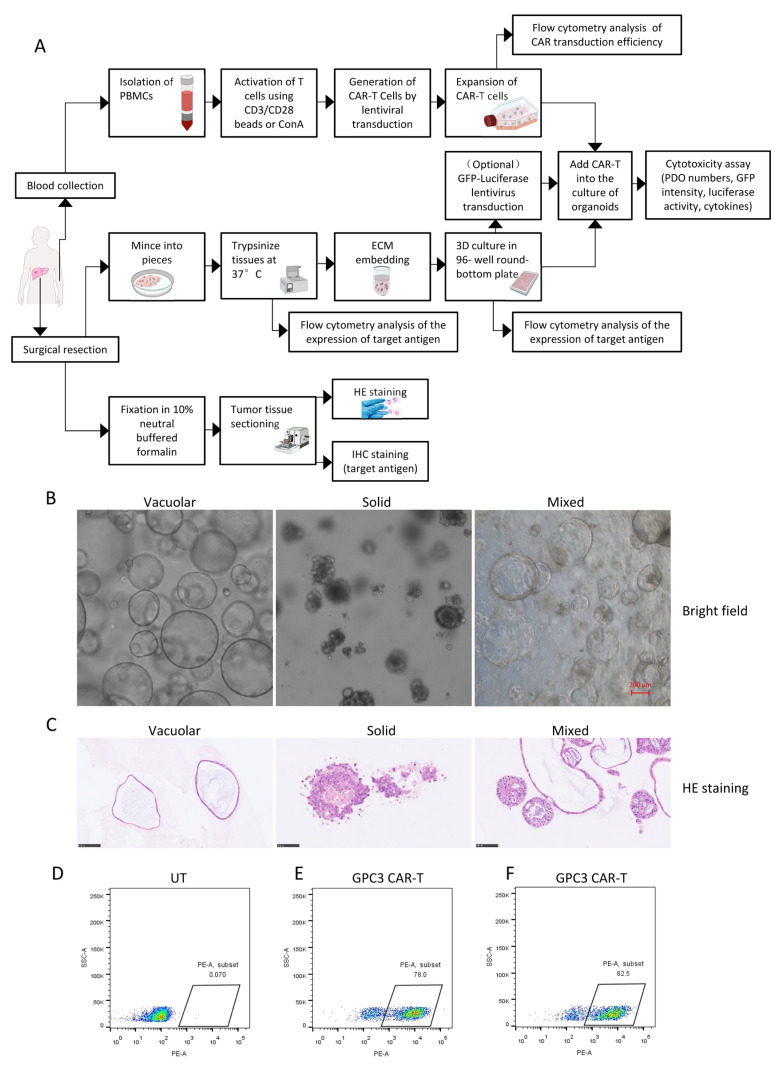
Establishment and characterization of a hepatocellular carcinoma (HCC) patient-derived organoid (PDO)-based platform for chimeric antigen receptor T cell (CAR-T) cell efficacy evaluation. (**A**) Schematic diagram of the CAR-T efficacy evaluation workflow based on HCC PDOs. (**B**) Bright-field images of representative patient-derived HCC organoids showing three predominant architectural patterns: vacuolar, solid, and mixed. Scale bar, 200 µm. (**C**) Hematoxylin and eosin (H&E)-stained sections of the three growth patterns illustrating the morphological heterogeneity of the organoids. Scale bar, 50 µm. (**D**) Untransduced T (UT) cells from the patient. (**E**,**F**) Transduction efficiency of CAR-T cells activated using CD3/CD28 beads (**E**) or Con-A (**F**).

We established a workflow to evaluate autologous GPC3-targeted CAR-T cells using patient-derived HCC organoids (PDOs; Figure 1A). HCC tissues and peripheral blood were collected from the same patient at the time of surgical resection. To generate organoids, tumor tissues were mechanically minced, enzymatically dissociated with collagenase I and collagenase IV, and embedded in Matrigel in 96-well round-bottom plates. After 7–10 days of culture, organoids were transduced with a lentiviral vector encoding GFP and luciferase. GPC3 expression was assessed at three stages: (1) in formalin-fixed HCC tissue sections by IHC, with H&E staining used to confirm tumor regions; (2) in freshly dissociated tumor cells before Matrigel embedding by flow cytometry; and (3) in established organoids by flow cytometry. In parallel, PBMCs isolated from the same patient were activated with CD3/CD28 beads or ConA and transduced with a CAR lentiviral vector to generate autologous CAR-T cells. After confirming CAR transduction efficiency, CAR-T cells were added to PDO cultures, and cytotoxicity was evaluated by organoid counting, luciferase activity, GFP intensity, and cytokine secretion.

Using freshly resected HCC specimens, we successfully established HCC PDOs with an overall success rate of approximately 80–90%. Bright-field imaging of representative organoids (Figure 1B) and H&E staining (Figure 1C) showed that the PDOs retained morphologic features comparable to those of the parental tumors. Based on morphology and histological architecture, HCC PDOs could be classified into three major subtypes: vacuolar, solid, and mixed (Figure 1B,C). Vacuolar organoids formed large cystic structures with a clear central lumen, solid organoids consisted of densely packed cellular aggregates, and mixed organoids displayed both cystic and solid components. These patterns closely resembled the architectural heterogeneity commonly observed in primary HCC.

To determine whether CAR-T cells could be generated from a minimal blood volume, PBMCs isolated from 5 mL of peripheral blood were activated with either CD3/CD28 beads (1:1) or 2.5 μg/mL concanavalin A (ConA), followed by transduction with a GPC3-h28Z lentiviral vector. Compared with UT cells (Figure 1D), GPC3 CAR expression reached 78.0% in CD3/CD28 bead-activated cells (Figure 1E) and 82.5% in ConA-activated cells (Figure 1F). These results indicate that GPC3 CAR-T cells can be generated with high transduction efficiency from a small volume of patient peripheral blood.

### 3.2. Patient-Derived HCC Organoids Recapitulate Key Features of the Primary Tumor

**Figure 2:**
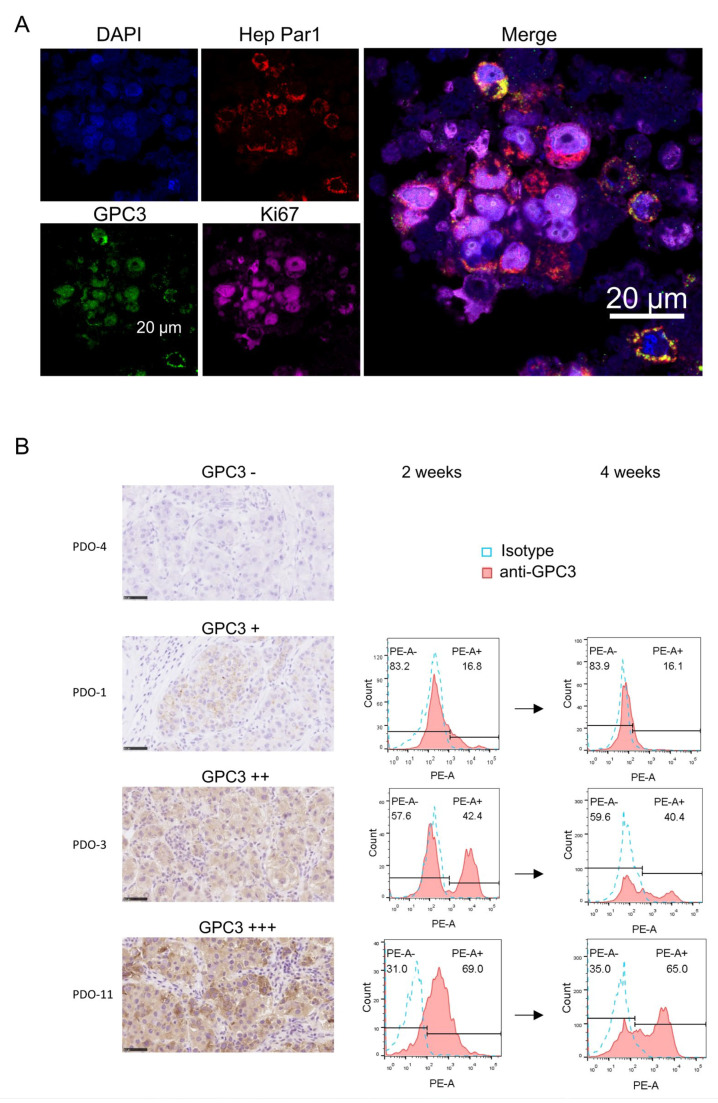
Patient-derived HCC organoids preserve key histologic and molecular features of the primary tumors. (**A**) Representative immunofluorescence staining of HCC organoids for Hep Par-1 (red), glypican-3 (GPC3, green), and Ki-67 (magenta), with DAPI nuclear counterstain (blue). Scale bar, 20 µm. (**B**) Representative GPC3 immunohistochemistry in primary HCC tissues (left panels) and corresponding flow cytometry histograms of GPC3 expression in matched PDOs after 2 weeks (middle panels) or 4 weeks (right panels) of culture. Cases are stratified as GPC3−, GPC3+, GPC3++, and GPC3+++ according to the percentage of positive tumor cells per field (average of multiple fields): − (0%), + (1–19%), ++ (20–50%), and +++ (>50%). Flow cytometry plots show anti-GPC3 staining (pink) overlaid with isotype control (blue). Scale bar, 50 µm.

To determine whether the established organoids faithfully preserved the biological characteristics of the original tumors, we examined their histologic and molecular features. Immunofluorescence staining showed strong expression of the hepatocytic marker Hep Par-1 in most cells, confirming their hepatocellular origin (Figure 2A). GPC3 was expressed in a substantial fraction of cells, and the proliferation marker Ki-67 showed widespread nuclear positivity, indicating active proliferation similar to that seen in the corresponding primary tumors (Figure 2A). These findings indicate that HCC PDOs preserve both the histologic architecture and molecular phenotype of the parental lesions.

Because target antigen expression is a key determinant of CAR-T efficacy, we next assessed whether the organoids reproduced the level and heterogeneity of GPC3 observed in primary HCC. IHC analysis of tumor tissues from four representative patients revealed pronounced intratumoral heterogeneity of GPC3 expression (Figure 2B). Consistently, flow cytometry of matched PDOs showed that GPC3 expression levels were largely maintained after 2 or 4 weeks of culture (Figure 2B). Collectively, these results indicate that the established HCC PDOs preserve key histologic and molecular features of the original tumors and support their use as a platform for functional evaluation of CAR-T responses [24].

The tumor microenvironment is another important determinant of CAR-T efficacy and, therefore, relevant to PDO-based testing. During organoid establishment, PDOs from a subset of patients retained abundant fibroblast-like stromal cells (Figure S1A). In addition, flow cytometry of two PDO cultures at different time points showed that a population of CD45^+^ immune cells persisted for approximately 1 month (Figure S1B). Because immune cell populations in organoid cultures gradually decline over time [22], HCC PDO-based cytotoxicity assays may be most informative when performed within the first month after establishment.

### 3.3. Matrigel Concentration Modulates the Growth and Transduction Efficiency of HCC PDOs

**Figure 3:**
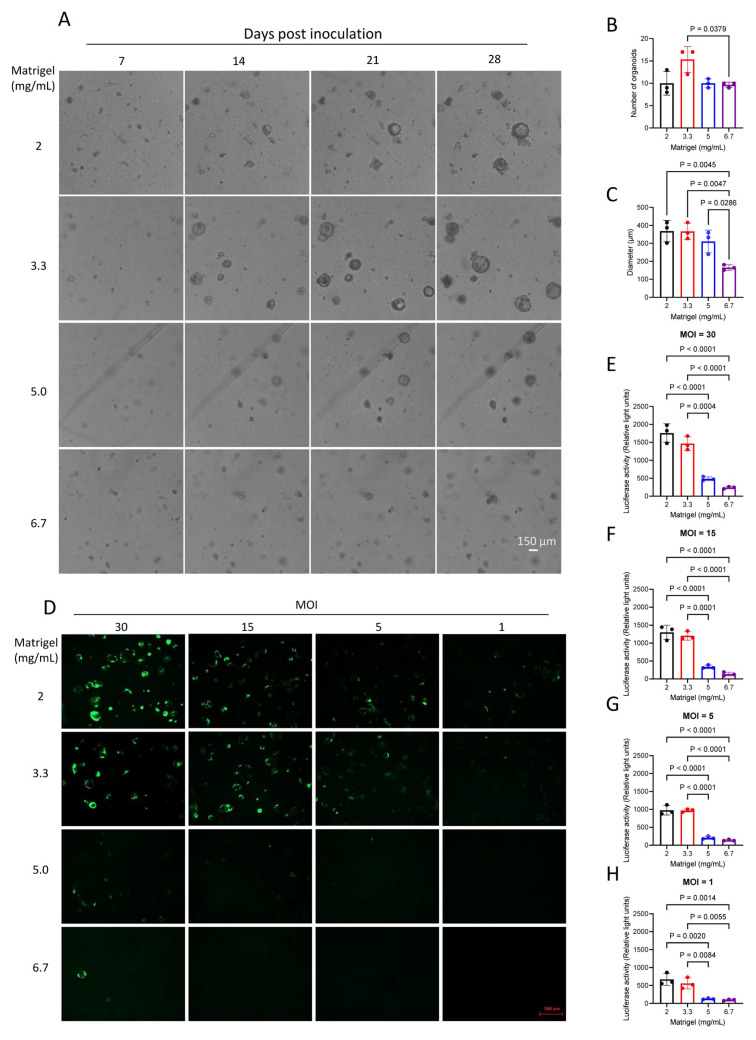
Matrigel concentration modulates the growth and transduction efficiency of HCC PDOs. (**A**) Representative bright-field images of HCC organoids embedded in Matrigel at final concentrations of 2, 3.3, 5, and 6.7 mg/mL and cultured for up to 28 days. Images were acquired at days 7, 14, 21, and 28 after inoculation, as indicated. Scale bars, 150 µm. (**B**) Quantification of organoid number under the indicated Matrigel concentrations. (**C**) Mean organoid diameter under the indicated Matrigel concentrations. (**D**) Representative fluorescence images of GFP-expressing HCC PDOs embedded in Matrigel at the indicated concentrations and infected with GFP–luciferase lentiviral vectors at multiplicities of infection (MOIs) of 30, 15, 5, and 1. Scale bar, 500 µm. (**E**–**H**) Luciferase activity of transduced PDOs cultured under the indicated Matrigel concentrations and infected at the indicated MOIs. Data are presented as mean ± SD (*n* = 3 independent wells per condition). *p* values were calculated by one-way analysis of variance (ANOVA) with multiple comparisons.

Because Matrigel plays a central role in supporting the three-dimensional organoid growth and maintaining phenotypic stability, we evaluated the effect of different Matrigel concentrations on HCC PDO growth. Cells obtained by tissue dissociation were seeded into 96-well round-bottom plates with different Matrigel concentrations. Organoids were imaged on days 7, 14, 21, and 28 (Figure 3A), and both organoid number and diameter were quantified. A Matrigel concentration of 3.3 mg/mL most effectively supported organoid growth, yielding the largest number of viable organoids with the greatest diameters (Figure 3B,C). This finding was reproduced in another independent PDO (Figure S2), indicating that 3.3 mg/mL is an optimal condition for primary HCC organoid culture. In contrast, a low Matrigel concentration (2 mg/mL) provided insufficient structural support and led to organoid collapse, whereas a high concentration (6.7 mg/mL) markedly restricted growth, resulting in fewer and smaller organoids (Figure 3B,C and Figure S2). Excessive Matrigel likely increases matrix stiffness, limits volumetric expansion, and impairs nutrient and waste diffusion, thereby reducing proliferation. These results indicate that intermediate-to-low Matrigel concentrations provide a better balance between structural support and organoid growth in HCC PDO culture.

Manual quantification of organoid growth was labor-intensive. Therefore, we explored reporter-based approaches using GFP and luciferase for rapid automated measurement. To identify optimal conditions for genetic manipulation, we systematically evaluated the effects of Matrigel concentration and viral dose on lentiviral transduction efficiency. Organoids cultured in Matrigel at 2, 3.3, 5, or 6.7 mg/mL were infected with a GFP–luciferase lentivirus at MOIs of 1, 5, 15, or 30. Fluorescence microscopy showed strong and widespread GFP expression in organoids cultured in low-to-intermediate Matrigel concentrations, particularly at 2–3.3 mg/mL, whereas GFP signals were markedly reduced in organoids embedded in 5 or 6.7 mg/mL Matrigel across all MOIs, suggesting that high matrix density limits lentiviral access and/or transduction efficiency (Figure 3D and Figure S3A).

Quantitative analyses using three independent readouts supported these observations (Figure 3E–H and Figure S3B,C). Across all MOIs, organoids cultured at 2–3.3 mg/mL exhibited significantly higher luciferase activity, GFP fluorescence intensity, and GFP-positive rates than those cultured at 5.0–6.7 mg/mL. The combination of lower Matrigel concentration (2–3.3 mg/mL) and higher MOI (15–30) produced the most robust and reproducible transduction, whereas higher Matrigel densities yielded near-background signals. Together, these findings indicate that Matrigel concentration is a critical determinant of efficient lentiviral gene delivery in 3D HCC organoids.

Notably, the Matrigel concentration that best supported organoid growth was not necessarily identical to the condition that most strongly facilitated viral transduction, underscoring the need to balance structural stability with experimental accessibility in PDO-based assays.

### 3.4. GPC3-Targeted CAR-T Cells Exhibit Clear Cytotoxicity Against HCC PDOs with High GPC3 Expression

**Figure 4:**
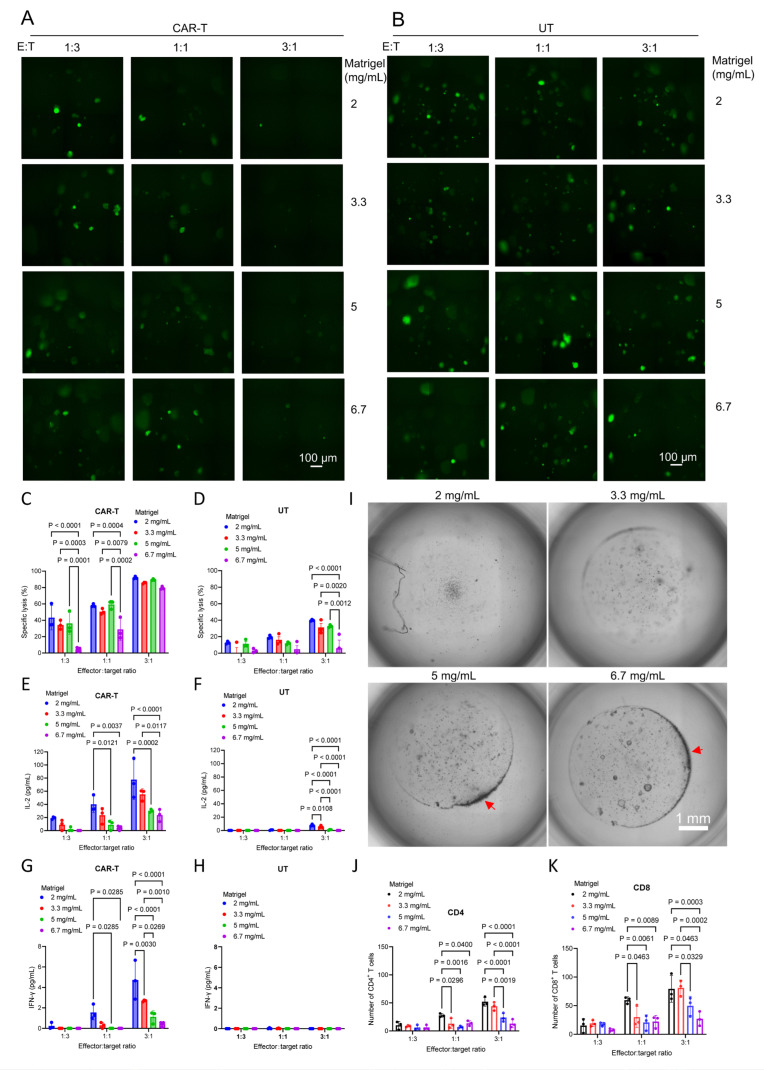
GPC3-targeted CAR-T cells exhibit clear cytotoxicity against HCC PDOs with high GPC3 expression. (**A**,**B**) Representative fluorescence images of GFP–luciferase–labeled HCC PDOs embedded in Matrigel at final concentrations of 2, 3.3, 5, and 6.7 mg/mL and co-cultured with either GPC3-targeted CAR-T cells (**A**) or UT cells (**B**) at effector-to-target (E:T) ratios of 1:3, 1:1, and 3:1, as indicated. Scale bar, 100 µm. (**C**,**D**) Specific lysis of GFP–luciferase–labeled HCC PDOs embedded in Matrigel at final concentrations of 2, 3.3, 5, and 6.7 mg/mL and co-cultured with either GPC3-targeted CAR-T cells (**C**) or UT cells (**D**) at E:T ratios of 1:3, 1:1, and 3:1, as indicated. (**E**,**F**) IL-2 concentrations in supernatants from PDO co-cultures with CAR-T cells (**E**) or UT (**F**). (**G**,**H**) IFN-γ concentrations in supernatants from PDO co-cultures with CAR-T (**G**) or UT cells (**H**). (**I**) Representative bright-field images showing the distribution and penetration of CAR-T cells in PDO-Matrigel cultures under different Matrigel concentrations. Scale bar, 1 mm. Red arrows indicate aggregated T cells. (**J**,**K**) Quantification of infiltrating CD4^+^ (**J**) and CD8^+^ (**K**) T cells recovered from PDO–Matrigel after co-culture at the indicated E:T ratios and Matrigel concentrations. Data are presented as mean ± SD (*n* = 3 independent wells per condition). Statistical significance was determined by two-way ANOVA with multiple comparisons.

To test the cytotoxicity of autologous CAR-T cells, we first used PDOs with high GPC3 expression. Because PDOs preserve key biological features of the original tumors, including antigen expression patterns, they represent a promising platform for functionally evaluating CAR-T activity. Although CAR-T cytotoxicity has been examined in organoid models, the optimal co-culture conditions remain incompletely defined. We, therefore, tested the impact of Matrigel concentration (2, 3.3, 5, and 6.7 mg/mL) on CAR-T-mediated killing. After confirming a CAR transduction efficiency of 42.1% by flow cytometry (Figure S4A), cytotoxicity assays were performed using GFP–luciferase–labeled, GPC3-high organoids. Organoids were dissociated, embedded in Matrigel, and seeded at 1000 cells per well in 96-well round-bottom plates. CAR-T cells or UT cells were added after gelation at the indicated effector-to-target ratios. Clear CAR-T-mediated cytotoxicity was observed by day 7 at E:T ratios of 3:1 and 1:1 (Figure 4A and Figure S4B), whereas UT cells showed minimal cytotoxicity under all conditions (Figure 4B and Figure S4C).

To quantify killing, we used three complementary readouts: integrated GFP fluorescence intensity, residual organoid number, and luciferase activity (Figure S5A–F). These metrics were concordant and showed that CAR-T cytotoxicity was strongly influenced by both Matrigel concentration and E:T ratio. Specifically, higher Matrigel concentrations and lower E:T ratios reduced killing efficiency (Figure S5B,D,F). Among the three assays, luciferase measurement was the most rapid and reproducible. At an E:T ratio of 3:1, UT cells caused a slight reduction in fluorescence and luciferase signals, indicating mild nonspecific suppression of organoid growth (Figure S5A,C,E).

Using luciferase activity to calculate specific lysis, CAR-T cells showed clear cytotoxicity at an E:T ratio of 3:1 across all Matrigel concentrations, whereas UT cells displayed only weak effects, most evident at low Matrigel concentrations (Figure 4C,D). Cytokine profiling of day-7 supernatants by CBA further revealed enhanced effector activation under low-Matrigel conditions. Both CAR-T and UT co-cultures produced higher IL-2 at lower Matrigel concentrations, whereas CAR-T cells secreted substantially more IFN-γ, which was undetectable in UT co-cultures (Figure 4E–H). TNF was detected only in CAR-T co-cultures at 2 mg/mL Matrigel with an E:T ratio of 3:1 (Figure S6). This elevated cytokine production corresponded with enhanced CAR-T cytotoxicity, indicating that lower Matrigel density promotes more effective CAR-T activation and tumor killing.

IL-6 was detected only in two wells under the highest Matrigel concentration (6.7 mg/mL): one UT co-culture (145.77 pg/mL; E:T = 3:1) and one CAR-T co-culture (119.8 pg/mL, E:T = 1:3). As IL-6 is typically produced by activated monocytes/macrophages [25,26], this finding suggests that residual non-tumor cellular components may persist in some PDO cultures and contribute to inflammatory cytokine production. However, the extent to which this system can model clinically relevant cytokine release phenomena remains unclear.

To further assess CAR-T infiltration, co-cultures were imaged on day 3. Matrigel concentration exerted a clear, concentration-dependent effect on T cell distribution. At higher concentrations (5–6.7 mg/mL), CAR-T cells mainly accumulated at the periphery and showed limited infiltration, whereas lower concentrations, particularly 2 mg/mL, allowed extensive penetration into the matrix (Figure 4I, entire culture well; Figure S7, magnified sectional view). Flow cytometry of cells recovered from Matrigel confirmed reduced CD8^+^ and CD4^+^ T cell infiltration with increasing Matrigel concentration and decreasing E:T ratio (Figure 4J,K). Together, these results indicate that matrix density is an important experimental determinant of CAR-T infiltration and cytotoxicity in HCC PDO co-cultures.

### 3.5. GPC3-Targeted CAR-T Cells Exhibit Modest Cytotoxicity Against HCC PDOs with Low GPC3 Expression

**Figure 5:**
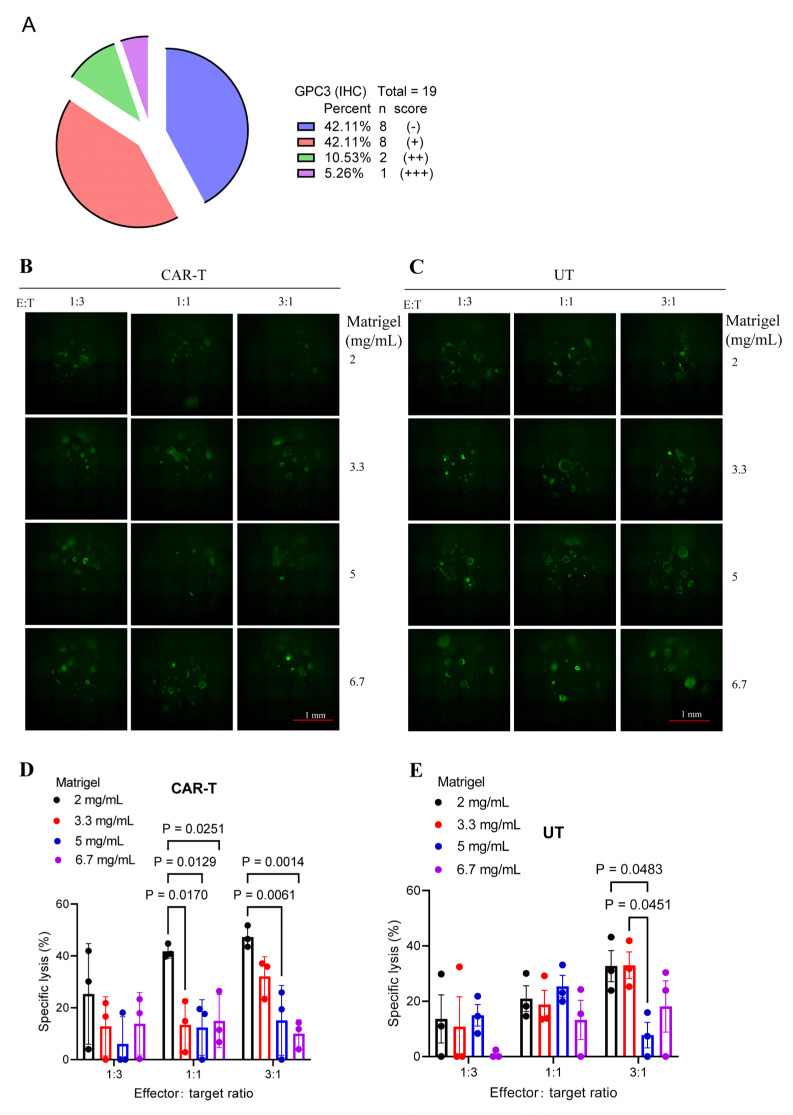
GPC3-targeted CAR-T cells exhibit modest cytotoxicity against HCC PDOs with low GPC3 expression. (**A**) GPC3 expression in tumor tissues from 19 patients with HCC as determined by IHC. GPC3 expression was scored semi-quantitatively based on the percentage of positive tumor cells per field (average of multiple fields): − (0%), + (1–19%), ++ (20–50%), and +++ (>50%). (**B**,**C**) Representative fluorescence images of GFP–luciferase–labeled HCC PDOs embedded in Matrigel at final concentrations of 2, 3.3, 5, and 6.7 mg/mL and co-cultured with either GPC3-targeted CAR-T cells (**B**) or UT cells (**C**) at E:T ratios of 1:3, 1:1, and 3:1. Scale bar, 100 µm. (**D**,**E**) Specific lysis of GFP–luciferase–labeled HCC PDOs co-cultured with either GPC3-targeted CAR-T cells (**D**) or UT cells (**E**) at the indicated Matrigel concentrations and E:T ratios.

We next assessed the prevalence of GPC3 expression in our HCC cohort by IHC analysis of tumor tissues from 19 patients. GPC3 was detected in 11 of 19 cases (58.9%); however, most positive tumors showed relatively low expression (8/11) (Figure 5A). To determine whether CAR-T cells remain effective against tumors with low target expression, we performed cytotoxicity assays using an HCC PDO with relatively low GPC3 levels (GPC3+). As observed in GPC3-high organoids, CAR-T-mediated killing decreased as Matrigel concentration increased (Figure 5B–E). However, compared with the GPC3-high PDOs shown in Figure 4C, CAR-T cells exhibited weaker cytotoxicity against GPC3-low organoids under the same Matrigel concentrations and E:T ratios (Figure 5D). These results support the notion that the efficacy of GPC3-targeted CAR-T cells is influenced by target antigen abundance.

### 3.6. Epigenetic Upregulation of GPC3 Sensitizes HCC PDOs to CAR-T Therapy

**Figure 6:**
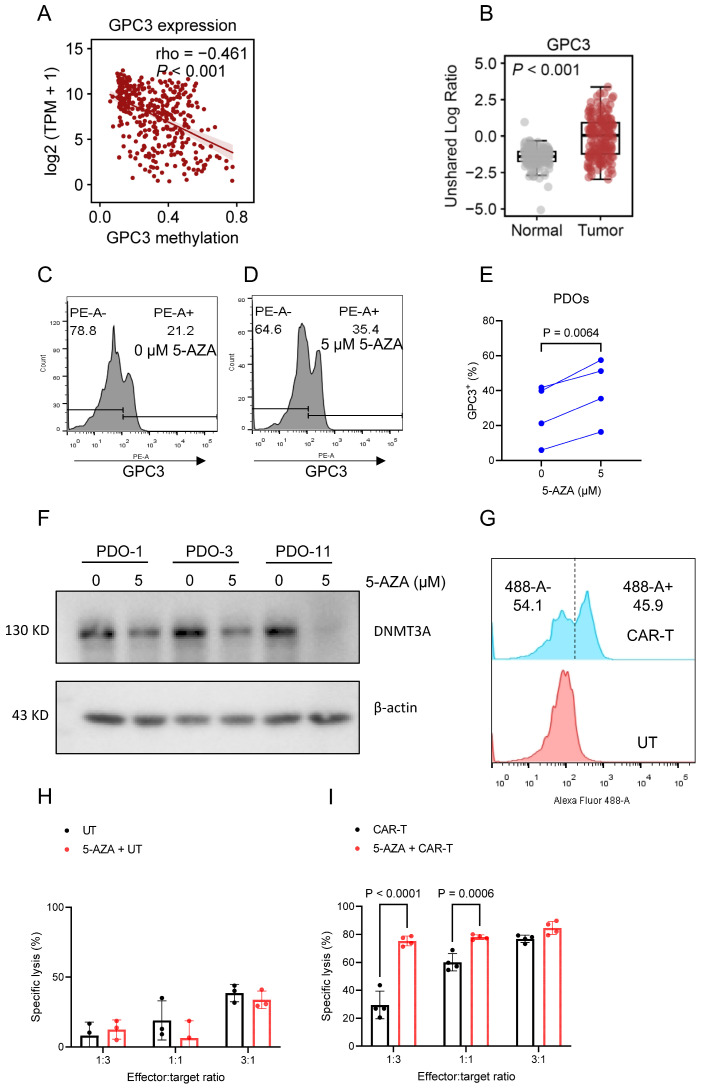
5-azacytidine (5-AZA) treatment increases GPC3 expression and enhances CAR-T-mediated killing in low-GPC3 HCC PDOs. (**A**) Relationship between GPC3 expression and promoter-region DNA methylation in HCC tumor tissues (*n* = 371) from The Cancer Genome Atlas (TCGA) HCC cohort. (**B**) GPC3 expression in HCC tumors (*n* = 165) and adjacent normal tissues (*n* = 165). (**C**,**D**) Representative flow-cytometry histograms of GPC3 expression in PDOs treated with vehicle (**C**) or 5 µM 5-AZA (**D**). (**E**) GPC3 expression in PDOs with or without treatment with 5 µM 5-AZA. Data are shown for four biologically independent PDO samples. (**F**) DNA methyltransferase 3 alpha (DNMT3A) expression in PDOs with or without treatment with 5 µM 5-AZA, as detected by western blotting. Data are shown for three biologically independent PDO samples. (**G**) CAR transduction efficiency of the prepared CAR-T cells. (**H**,**I**) Specific lysis of PDOs by UT (**H**) or GPC3-targeted CAR-T cells (**I**) with or without 5-AZA pretreatment of PDOs at the indicated E:T ratios. Data are presented as mean ± SD (*n* = 3 independent wells per condition). *p* values were calculated by two-way ANOVA with multiple comparisons.

Given the marked heterogeneity of GPC3 expression in HCC and its strong impact on CAR-T efficacy, we investigated whether increasing GPC3 expression could enhance CAR-T activity. Analysis of the TCGA cohort suggested a negative correlation between GPC3 expression and promoter methylation (Figure 6A), consistent with a previous study [27]. We also examined GPC3 protein expression in 165 pairs of HCC tumor tissues and adjacent normal tissues and found that GPC3 protein was significantly higher in tumor tissues than in matched normal tissues (Figure 6B). However, a subset of tumors showed relatively low GPC3 expression.

We, therefore, treated four low-GPC3 PDO lines with 5 μmol/L 5-azacytidine (5-AZA), a DNA methyltransferase inhibitor. Flow cytometry showed that 5-AZA treatment increased GPC3 expression in all four organoid lines (Figure 6C–E). Further analysis confirmed that 5-AZA significantly reduced the expression of DNMT3A, an enzyme involved in DNA methylation (Figure 6F). After generating CAR-T cells and confirming a transduction efficiency of 45.9% by flow cytometry (Figure 6G), we evaluated CAR-T cytotoxicity using a luciferase-based assay at three E:T ratios. Pretreatment with 5-AZA significantly enhanced CAR-T-mediated killing compared with CAR-T alone at E:T ratios of 1:3 and 1:1, indicating that 5-AZA treatment can enhance the sensitivity of low-GPC3 HCC PDOs to CAR-T-mediated killing (Figure 6H,I). These results indicate that 5-AZA can increase the sensitivity of low-GPC3 HCC PDOs to GPC3-targeted CAR-T cell killing. More broadly, these findings support the use of HCC organoids as a functional platform for evaluating CAR-T activity and for exploring combination approaches, including epigenetic modulation.

## 4. Discussion

Despite substantial progress in immunotherapy, the clinical efficacy of CAR-T cell therapy in solid tumors, including HCC, remains limited and highly variable [4,6,7]. A central challenge is the lack of preclinical models that can faithfully capture patient-specific tumor features while enabling functional evaluation of immune cell activity [28]. In this study, we developed a patient-derived organoid (PDO)-based platform that integrates tumor-intrinsic heterogeneity with functional immunologic assays to evaluate GPC3-targeted CAR-T cell responses. Our findings indicate that this system preserves therapeutically relevant antigen heterogeneity, supports functional assessment of CAR-T sensitivity, and provides a framework for exploring strategies to overcome antigen-low resistance.

A key insight from this study is that antigen abundance acts as a quantitative determinant of CAR-T efficacy in HCC. By leveraging PDOs that retain heterogeneous GPC3 expression patterns, our data support a relationship between CAR-T-mediated cytotoxicity and target antigen density. Organoids with high GPC3 expression were efficiently eradicated, whereas those with low or heterogeneous expression showed markedly reduced sensitivity. These findings provide functional evidence supporting antigen heterogeneity as a major driver of resistance in solid tumor CAR-T therapy [29]. Importantly, this relationship was observed in a patient-derived system, strengthening its translational relevance compared with conventional cell line-based models.

Beyond tumor-intrinsic antigen variability, our study highlights the strong influence of matrix conditions on measured CAR-T function in this organoid system. We found that Matrigel concentration exerted a clear, concentration-dependent effect on CAR-T cell infiltration, spatial distribution, and cytotoxic activity. Higher matrix density restricted T cell penetration and reduced killing efficiency, whereas lower concentrations permitted robust infiltration and effector function [30]. These findings are consistent with emerging evidence that the physical properties of the tumor microenvironment, including matrix stiffness and density, can act as barriers to immune cell trafficking and function [30,31]. Importantly, our data indicate that matrix parameters can substantially influence experimental outcomes in organoid-based assays, underscoring the need for standardized and physiologically relevant conditions when evaluating immunotherapies in vitro.

Functionally, the PDO-based co-culture system enabled integrated assessment of CAR-T activity through multiple complementary readouts, including cytotoxicity, cytokine secretion, and immune-cell infiltration. The concordance among these measures supports the robustness of the platform. Notably, cytokine profiling revealed that effective tumor killing was associated with increased production of effector cytokines such as IFN-γ and IL-2, consistent with canonical CAR-T activation [29]. In contrast, minimal cytokine release was observed under conditions associated with limited cytotoxicity, further reinforcing the functional relevance of the system. The occasional detection of IL-6 suggests that residual myeloid components may persist in some PDO cultures and contribute to inflammatory signaling; however, whether this model can recapitulate clinically relevant toxicity, such as cytokine release syndrome, warrants further investigation.

Importantly, our study identifies antigen plasticity as a therapeutically exploitable mechanism to overcome resistance. We demonstrate that GPC3 expression in HCC PDOs can be increased by treatment with the DNA methyltransferase inhibitor 5-AZA. Further analysis showed that 5-AZA reduced the expression of DNMT3A, an enzyme involved in DNA methylation, suggesting that 5-AZA may enhance GPC3 expression, at least in part, through epigenetic regulation. This pharmacologic modulation significantly enhanced CAR-T-mediated cytotoxicity in GPC3-low organoids, supporting a functional link between increased antigen expression and improved therapeutic response. These findings suggest that some antigen-low tumors may remain susceptible to modulation and may not be irreversibly resistant to CAR-T therapy. This concept provides a strong rationale for combination strategies that increase target antigen density to expand the population of patients who may benefit from CAR-T therapy.

Collectively, these results support the potential of PDO-based functional testing for patient-specific evaluation of CAR-T therapy. Compared with traditional cell lines and patient-derived xenograft models, PDOs offer several advantages, including preservation of tumor heterogeneity, compatibility with autologous immune cells, and relatively rapid turnaround time [29,32]. Importantly, this platform enables direct functional interrogation of CAR-T activity in a patient-relevant context, which may complement existing biomarker-based approaches [29,32,33]. While further validation is required, particularly in prospective clinical settings, our findings support the potential of PDO-based assays to complement biomarker-based approaches and to guide future evaluation of combination strategies.

Artificial intelligence (AI)-assisted analysis may further enhance the utility and efficiency of PDO-based CAR-T evaluation platforms in the future. PDO models can generate multidimensional datasets, including organoid morphology, growth kinetics, target antigen expression, CAR-T infiltration and cytotoxicity, cytokine release profiles, and potentially genomic, transcriptomic, epigenetic, and clinical information. AI-based approaches could help integrate these heterogeneous data types and identify predictive patterns associated with CAR-T responsiveness or resistance. AI has already been explored for early diagnosis, predictive modeling, surgical decision support, and precision medicine, supporting its potential application in personalized oncology [34]. In the context of PDO-based CAR-T testing, machine learning may be used to analyze organoid imaging data, quantify dynamic treatment responses, and predict which patients are more likely to benefit from GPC3-targeted CAR-T therapy. AI-assisted modeling may also help optimize culture conditions, E:T ratios, matrix composition, and combination strategies. Although validation in larger patient cohorts and standardized datasets is still required, integrating AI with PDO-based functional testing may provide a promising strategy to improve the precision and scalability of personalized CAR-T therapy for HCC.

Several limitations of this study should be acknowledged. First, the sample size was relatively limited, particularly for the key functional CAR-T assays. Therefore, broader validation using a larger cohort of HCC PDOs with diverse GPC3 expression levels will be necessary to confirm the generalizability of our findings. In addition, interpatient variability in CAR-T cell products may influence functional readouts and was not comprehensively addressed in the current study. Second, although PDOs retain key tumor-intrinsic features and antigen heterogeneity, they do not fully recapitulate the complexity of the in vivo tumor microenvironment. Non-malignant components, including immune and stromal cells, may vary among PDO lines and gradually decline during culture, which may limit the assessment of long-term immune-tumor interactions. Third, our co-culture assays were performed over a relatively short period and, therefore, may not fully reflect long-term CAR-T cell persistence, exhaustion, or sustained antitumor activity. Fourth, although the matrix conditions used in this study provided important insights into organoid growth, viral transduction, and CAR-T infiltration, Matrigel-based systems may not fully reproduce the extracellular matrix composition and mechanical properties of native HCC tissues. Fifth, while our data highlight the importance of GPC3 expression in determining CAR-T sensitivity, the broader molecular mechanisms underlying CAR-T resistance remain incompletely defined. Further studies integrating genomic, transcriptomic, epigenetic, and immune profiling may help identify additional resistance mechanisms and predictive biomarkers. Finally, although 5-AZA increased GPC3 expression and enhanced CAR-T sensitivity in GPC3-low PDOs, its broader effects on tumor biology, off-target gene regulation, and immune interactions require further investigation. Future studies using larger patient cohorts, standardized PDO–CAR-T testing protocols, in vivo validation, and clinical outcome correlation will be important to establish the translational value of this platform.

In summary, we established a PDO-based platform for functional evaluation of GPC3-targeted CAR-T responses and provided proof-of-concept evidence supporting antigen-dependent resistance in HCC. Our findings highlight antigen density as an important determinant of CAR-T efficacy and suggest that epigenetic modulation can sensitize antigen-low tumors to CAR-T-mediated killing. These results support a model in which CAR-T therapy in solid tumors may be optimized through both patient-specific functional evaluation and rational combination strategies. With further validation, this approach may contribute to improving the precision and efficacy of CAR-T therapy in HCC.

## 5. Conclusions

This study establishes a patient-derived organoid–based platform for the functional evaluation of GPC3-targeted CAR-T cell therapy in HCC. By preserving tumor-specific antigen heterogeneity and enabling direct co-culture with autologous CAR-T cells, this system provides a framework for assessing patient-specific features relevant to therapeutic sensitivity. Our findings identify antigen density as an important determinant of CAR-T efficacy and suggest that epigenetic modulation can sensitize antigen-low tumors to CAR-T-mediated killing. In addition, we show that matrix conditions critically influence CAR-T infiltration and cytotoxic activity, underscoring the importance of microenvironmental factors in shaping therapeutic outcomes.

Together, these results support a model in which effective CAR-T therapy in solid tumors requires both adequate target antigen expression and a permissive microenvironment for immune-cell access. By enabling functional evaluation of CAR-T responses and exploration of combination strategies, this organoid-based platform provides a foundation for improving the precision and efficacy of CAR-T therapy in HCC. Further validation in clinical settings will be essential to determine its utility in guiding patient selection and treatment optimization.

## Data Availability

The original contributions presented in this study are included in the article/Supplementary Materials. Further inquiries can be directed to the corresponding authors.

## Supplementary Materials

The following supporting information can be downloaded at: https://www.mdpi.com/article/10.3390/cells15090799/s1, Table S1: HCC PDOs culture media; Figure S1: Patient-derived HCC organoids retain abundant fibroblast-like stromal cells and immune cells; Figure S2: The growth of Patient-derived HCC organoid embedded in at different concentrations Matrigel; Figure S3: Optimization of Matrigel concentration and viral MOI for efficient lentiviral transduction of HCC organoids; Figure S4: CAR-T cytotoxicity against HCC organoids under different Matrigel concentration; Figure S5: CAR-T cytotoxicity against HCC organoids under different Matrigel concentration; Figure S6: Cytokine profiling of CAR-T-mediated killing of HCC organoids; Figure S7: Impact of Matrigel concentration on CAR-T cell penetration.

## Abbreviations

The following abbreviations are used in this manuscript:

5-AZA	5-azacytidine
ANOVA	One-way analysis of variance
CAR-T	Chimeric antigen receptor T cell
CBA	Cytometric bead array
DMEM	Dulbecco’s modified Eagle medium
DNMT3A	DNA methyltransferase 3 alpha
E:T	Effector-to-target
GDC	Genomic data commons
GPC3	Glypican-3
HCC	Hepatocellular carcinoma
MOI	Multiplicity of infection
PDO	Patient-derived organoid
TCGA	The cancer genome atlas
TME	Tumor microenvironment
TPM	Transcripts per million
UT	Untransduced T
