# Supplementary material for: Patient-Derived Organoid Modeling of Glypican-3 CAR-T Responses in Hepatocellular Carcinoma
Source: Cells. 2026 Apr 28;15(9):799. doi: 10.3390/cells15090799 (PMC13163092; doi:10.3390/cells15090799)
Supplement: Supplementary file 1 [file cells-15-00799-s001.zip › cells-4269070-supplementary.pdf]

Supplementary Materials

Supplementary Materials

HCC PDOs culture media

Human HCC PDOs were cultured in Advanced DMEM/F12 medium, containing R-spondin 1, Noggin, Wnt-3a, EGF, HEPES, Glutamax, N2, B27, n-Acetylcysteine, Normocin, Penicillin-Streptomycin, Niacinamide, Gastrin, Prostaglandin E2, A83-01, SB202190, FGF, Forskolin, TGFa, Dexamethasone, HGF, Minocycline hydrochloride. More detailed information is shown in Supplemental Table S1.

Table S1. HCC PDOs culture media

	Regent name	Company	Cat No.	Stock solution	Solvent	Final concentration
1	Advanced DMEM/F12	Gibco	12634-010	/	1×	1×
2	R-spondin1	Sino Biological Inc.	11083-HNAS	50 µg/ mL	0.1%BSA/PBS	500 ng/mL
3	Noggin	Sino Biological Inc.	10267-HNAH	10 µg/ mL	0.1%BSA/PBS	100 ng/mL
4	Wnt-3a	ProteinTech	HZ-1296	10 µg	0.1%BSA/PBS	100 ng/mL
5	Human EGF	Sino Biological Inc.	50482-MNCH	500 µg/ mL	0.1%BSA/PBS	80 ng/mL
6	HEPES	Gibco	15630080	100 ×	/	1×
7	Glutamax	Gibco	35050061	100 ×	/	1×
8	N2	Invitrogen	17502-048	50 ×	/	1×
9	B27	Invitrogen	17504-044	100 ×	/	1×
10	n-Acetylcysteine	Sigma-aldrich	A9165	500 mM	ddH_2O	1.5 mM
11	Penicillin-Streptomycin	Gibco	15140122	100 ×	/	1×
12	Niacinamide	Sigma-aldrich	N0636	400 mM	ddH_2O	10 mM
13	Gastrin	Sigma-aldrich	G9145	100 µM	0.1%BSA/PBS	15 nM
14	Prostaglandin E2	Sigma-aldrich	P6532/P5640-1mg	2.837 mM	DMSO	1 µM
15	A83-01	Tocris/Sigma	2939/SML0788	5 mM	DMSO	2 µM
16	SB202190	Sigma-aldrich	S7067	6 mM	DMSO	3 µM
17	FGF	Peprotech	100-18B-100ug	60 ng/mL	0.1%BSA/PBS	60 ng/mL
18	Forskolin	TargetMol	T2939 50 mg	100 mM	DMSO	10 µM
19	TGFa	Peprotech	100-16A-100ug	1 mg/mL	0.1%BSA/PBS	30 ng/mL
20	Dexamethasone	Beyotime	ST1254-250mg	3 µM	ddH_2O	3 nM
21	HGF	Peprotech	100-39-10UG	10µg/ mL	0.1%BSA/PBS	10 ng/mL
22	Minocycline hydrochloride	TargetMol	T1101-100 mg	10mg/mL，5000x	ddH_2O	1x

A

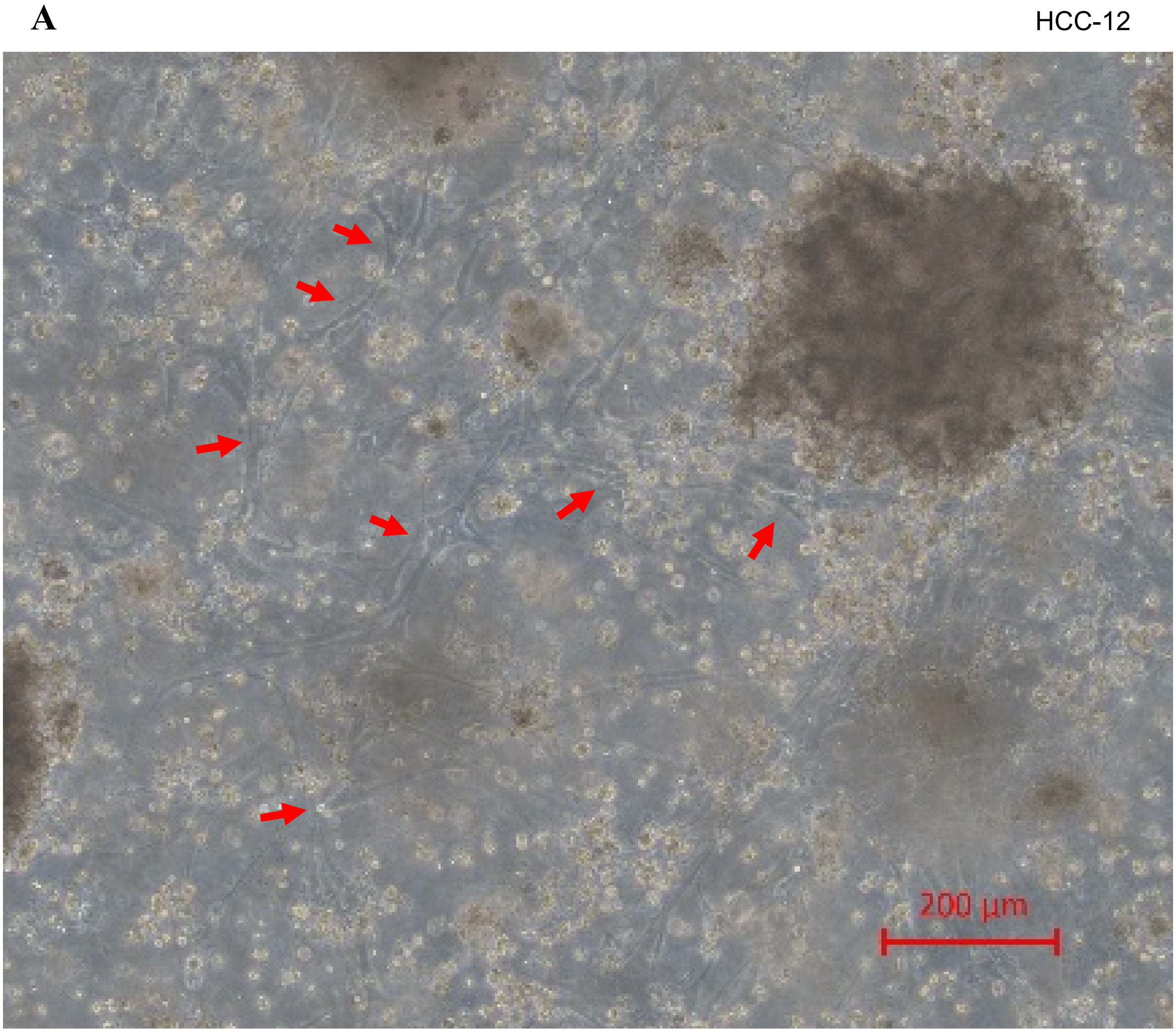

B

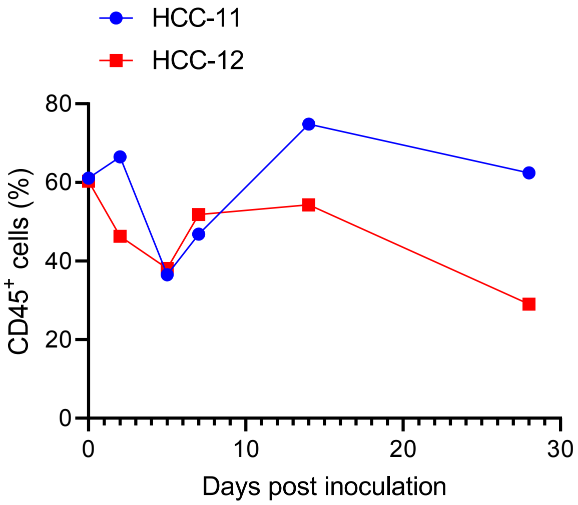

Figure S1. Patient-derived HCC organoids retain abundant fibroblast-like stromal cells and immune cells. (A) Bright-field image of representative organoid showing fibroblast-like stromal cells (red arrow). Scale bar, 200 µm. (B) Percentage of $CD45^+$ immune cells in two distinct organoid cultures identified on day 0, 2, 5, 7, 14 and 28 post-inoculation by flow cytometry.

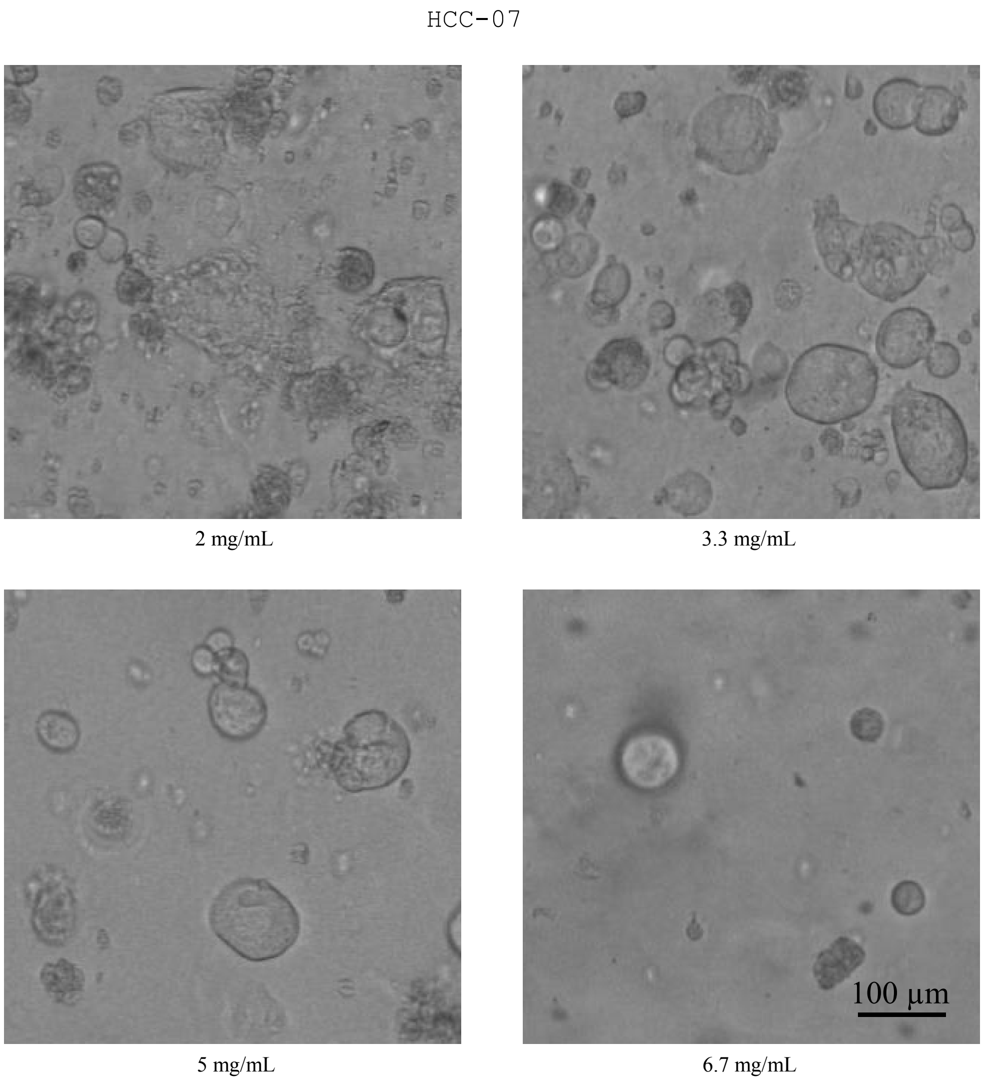

Figure S2. The growth of Patient-derived HCC organoid embedded in at different concentrations Matrigel. Representative bright-field images of HCC organoids embedded in Matrigel at final concentrations of 2, 3.3, 5 and 6.7 mg/mL and cultured for 28 days. Scale bar, 100 µm.

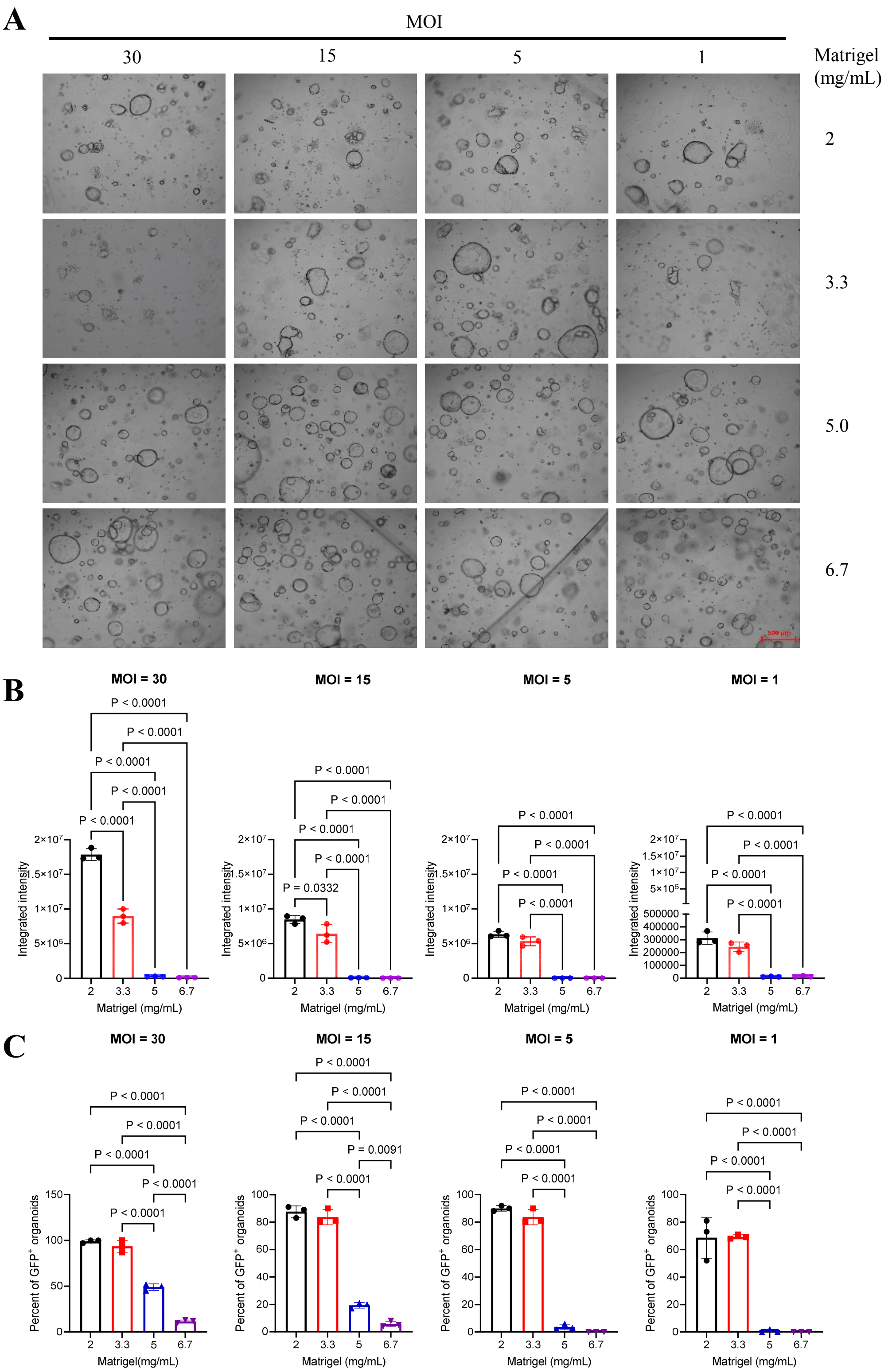

Figure S3. Optimization of Matrigel concentration and viral MOI for efficient lentiviral transduction of HCC organoids. (A) Representative bright-field images of HCC PDOs embedded in Matrigel at final concentrations of 2, 3.3, 5 and 6.7 mg/mL and infected with GFP–luciferase lentiviral vectors at the indicated multiplicity of infection (MOI = 30, 15, 5 and 1). Scale bar, 500 μm. (B) Quantification of GFP fluorescence intensity (integrated density per well) in PDOs cultured in Matrigel at the indicated concentrations for each MOI. (C) Percentage of GFP^{+} organoids among total organoids under the indicated conditions. Data are presented as mean ± SD (n = 3 independent wells per condition). P values were calculated by one-way ANOVA with multiple comparisons.

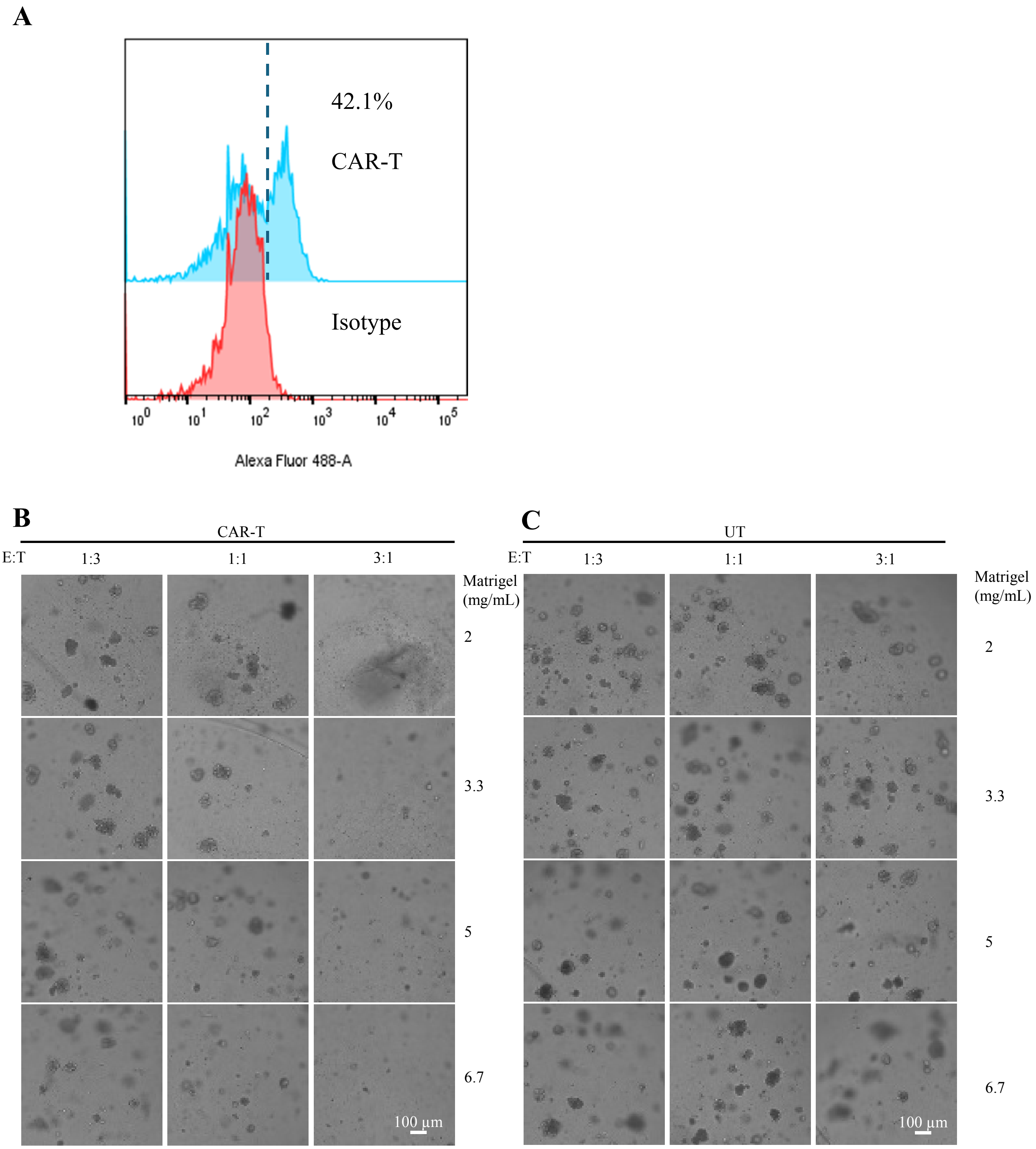

Figure S4. CAR-T cytotoxicity against HCC organoids under different Matrigel concentration. (A) The CAR transduction efficiency determined by flow cytometry. (B and C) Representative bright-field images of GFP–luciferase–labeled HCC PDOs embedded in Matrigel at final concentrations of 2, 3.3, 5 and 6.7 mg/mL and co-cultured with either GPC3-targeted CAR-T cells (B) or UT cells (C) at effector:target (E:T) ratios of 1:3, 1:1 and 3:1, as indicated. Scale bar, 100 µm.

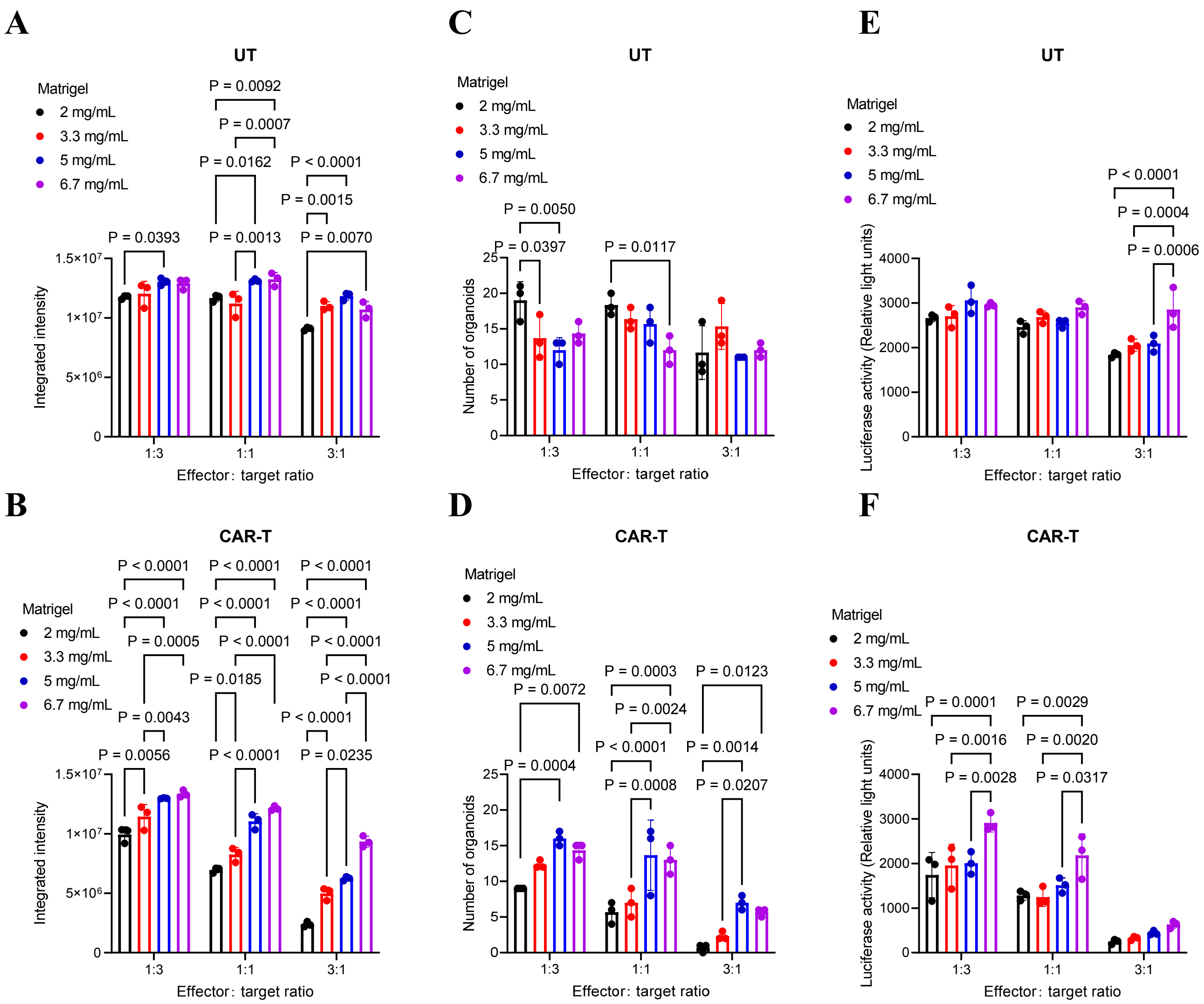

Figure S5. CAR-T cytotoxicity against HCC organoids under different Matrigel concentration. GFP–luciferase–labeled HCC PDOs embedded in Matrigel at final concentrations of 2, 3.3, 5 and 6.7 mg/mL and co-cultured with either GPC3-targeted CAR-T cells or UT cells at effector:target (E:T) ratios of 1:3, 1:1 and 3:1, as indicated. (A and B) Quantification of organoid GFP fluorescence (integrated intensity) after co-culture with UT (A) or CAR-T cells (B) under the indicated conditions. (C and D) Numbers of residual organoids after co-culture with UT (C) or CAR-T cells (D). (E and F) Luciferase activity of PDOs co-cultured with UT (E) or CAR-T cells (F) at the indicated Matrigel concentrations and E:T ratios. Data in (A–F) are presented as mean ± SD (n = 3 independent wells per condition). Statistical significance was determined by two-way ANOVA with multiple comparisons.

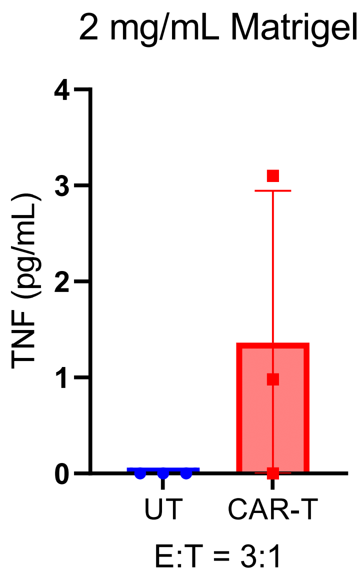

Figure S6. Cytokine profiling of CAR-T–mediated killing of HCC organoids. Concentrations of TNF in supernatants from PDO co-cultures with CAR-T or UT cells. Data are presented as mean ± SD (n = 3).

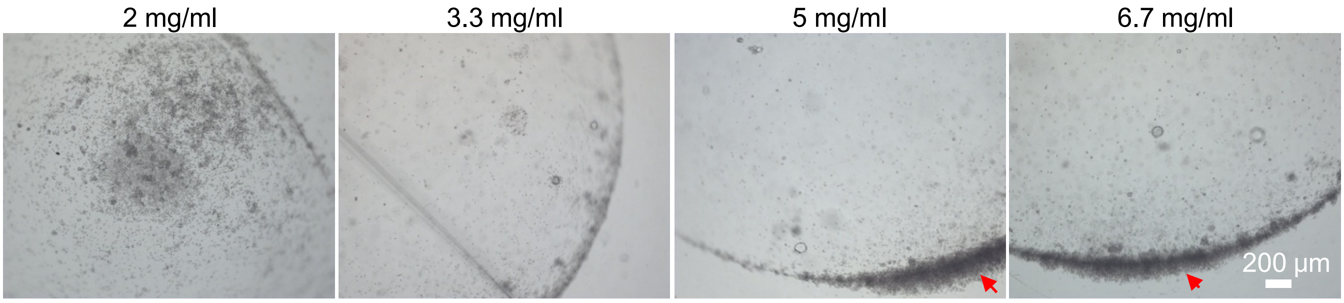

Figure S7. Impact of Matrigel concentration on CAR-T cell penetration. Representative bright-field images of CAR-T cells co-cultured with PDOs embedded in Matrigel at final concentrations of 2, 3.3, 5 and 6.7 mg/mL, illustrating the distribution and penetration of T cells within Matrigel of different concentration (Scale bar, 200 µm). Red arrows point to aggregated T cells.
